# Direct detection and characterization of foot‐and‐mouth disease virus in East Africa using a field‐ready real‐time PCR platform

**DOI:** 10.1111/tbed.12684

**Published:** 2017-07-30

**Authors:** E. L. A. Howson, B. Armson, N. A. Lyons, E. Chepkwony, C. J. Kasanga, S. Kandusi, N. Ndusilo, W. Yamazaki, D. Gizaw, S. Cleaveland, T. Lembo, R. Rauh, W. M. Nelson, B. A. Wood, V. Mioulet, D. P. King, V. L. Fowler

**Affiliations:** ^1^ The Pirbright Institute Pirbright Surrey UK; ^2^ Institute of Biodiversity, Animal Health and Comparative Medicine College of Medical Veterinary & Life Sciences University of Glasgow Glasgow UK; ^3^ European Commission for the Control of Foot‐and‐Mouth Disease (EuFMD) Animal Production and Health Division FAO Rome Italy; ^4^ Foot‐and‐Mouth Disease Laboratory, Embakasi Ministry of Agriculture, Livestock, Fisheries and Blue Economy Nairobi Kenya; ^5^ Department of Biochemistry, Molecular Biology and Biotechnology College of Veterinary and Medical Sciences Sokoine University of Agriculture, Chuo Kikuu Morogoro Tanzania; ^6^ Department of Veterinary Science Faculty of Agriculture University of Miyazaki Miyazaki Japan; ^7^ National Animal Health Diagnostic & Investigation Centre Sebeta Oromia Ethiopia; ^8^ Tetracore Inc Rockville MD USA

**Keywords:** diagnostics, disease control, foot‐and‐mouth disease, foot‐and‐mouth disease virus, lyophilized, rRT‐PCR

## Abstract

Effective control and monitoring of foot‐and‐mouth disease (FMD) relies upon rapid and accurate disease confirmation. Currently, clinical samples are usually tested in reference laboratories using standardized assays recommended by The World Organisation for Animal Health (OIE). However, the requirements for prompt and serotype‐specific diagnosis during FMD outbreaks, and the need to establish robust laboratory testing capacity in FMD‐endemic countries have motivated the development of simple diagnostic platforms to support local decision‐making. Using a portable thermocycler, the T‐COR™ 8, this study describes the laboratory and field evaluation of a commercially available, lyophilized pan‐serotype‐specific real‐time RT‐PCR (rRT‐PCR) assay and a newly available FMD virus (FMDV) typing assay (East Africa‐specific for serotypes: O, A, Southern African Territories [SAT] 1 and 2). Analytical sensitivity, diagnostic sensitivity and specificity of the pan‐serotype‐specific lyophilized assay were comparable to that of an OIE‐recommended laboratory‐based rRT‐PCR (determined using a panel of 57 FMDV‐positive samples and six non‐FMDV vesicular disease samples for differential diagnosis). The FMDV‐typing assay was able to correctly identify the serotype of 33/36 FMDV‐positive samples (no cross‐reactivity between serotypes was evident). Furthermore, the assays were able to accurately detect and type FMDV RNA in multiple sample types, including epithelial tissue suspensions, serum, oesophageal–pharyngeal (OP) fluid and oral swabs, both with and without the use of nucleic acid extraction. When deployed in laboratory and field settings in Tanzania, Kenya and Ethiopia, both assays reliably detected and serotyped FMDV RNA in samples (*n* = 144) collected from pre‐clinical, clinical and clinically recovered cattle. These data support the use of field‐ready rRT‐PCR platforms in endemic settings for simple, highly sensitive and rapid detection and/or characterization of FMDV.

## 1 INTRODUCTION

Early detection of foot‐and‐mouth disease virus (FMDV), a highly infectious picornavirus, is essential to minimize the impacts of foot‐and‐mouth disease (FMD) in susceptible populations. FMD is endemic in many countries throughout Africa, Asia and parts of South America, existing as seven distinct FMDV serotypes (A, O, C, Asia 1, Southern African Territories [SAT] 1, 2 and 3), which are distributed unevenly worldwide within seven geographically defined pools (Paton, Sumption, & Charleston, 2009). Laboratory‐based diagnostic tests play an essential role in FMD control and eradication by providing accurate confirmation of clinical signs. Real‐time reverse‐transcription polymerase chain reaction (rRT‐PCR) has been widely adopted by FMD reference laboratories as a principal tool for FMDV detection, offering high analytical sensitivity and rapid sample throughput (Reid et al., 2009; Shaw et al., 2007). Pan‐serotype‐specific rRT‐PCR assays, targeting conserved regions of the FMDV RNA genome (Callahan et al., 2002; Reid et al., 2002), were used during the UK 2007 FMDV outbreak to test 99.1% of 3,246 diagnostic samples submitted to the UK National Reference Laboratory for FMD (The Pirbright Institute, UK) (Reid et al., 2009).

Although laboratory‐based tests provide rapid and accurate results, sample transportation to the laboratory can negatively affect the quality of the specimens (if shipped incorrectly) and delay/or even hinder the processes of immediate critical decision‐making and disease control. Furthermore, many diagnostic procedures require sophisticated and expensive equipment, limiting their application in low‐ and middle‐income countries (LMICs), which lack the infrastructure and financial resources for veterinary diagnostics and surveillance of endemic diseases such as FMD (Namatovu et al., 2013; Paton et al., 2009; Vosloo, Bastos, Sangare, Hargreaves, & Thomson, 2002). The control of FMD has been recognized by the Food and Agricultural Organization of the United Nations (FAO) and The World Organisation for Animal Health (OIE) as a global priority (Sumption, Domenech, & Ferrari, 2012). However, with effective control strategies requiring knowledge of FMD distribution and epidemiology, the deployment of simple point‐of‐care test (POCT) platforms for active FMDV detection, monitoring and characterization remains an ongoing research effort.

The compatibility of an OIE‐recommended rRT‐PCR assay with POCT platforms has already been demonstrated (Hearps, Zhang, & Alexandersen, 2002; Madi et al., 2012; Monwina, Clavijo, Li, Collignon, & Kitching, 2007; Paixão et al., 2008). For instance, portable rRT‐PCR has been deployed in field settings for accurate detection of FMDV in serum, epithelial suspensions and oesophageal–pharyngeal fluid (OP) fluid (Howson et al., 2015). However, current assay formats and platforms are currently limited by low sample throughput, the requirement for RNA extraction (and thus methods and equipment to perform this) or are commercially unavailable. Furthermore, evaluation of POCT rRT‐PCR platforms has only been performed using FMDV pan‐serotype‐specific assay formats. With methods of FMD control in endemic settings relying upon rapid and accurate identification of the particular FMDV serotype circulating in the field (e.g. targeted vaccination) (Sumption et al., 2012), there have been efforts to design serotype‐specific assays (tailored to the region) that target variable capsid‐coding regions of the FMDV genome (Ahmed et al., 2012; Bachanek‐Bankowska et al., 2016; Giridharan, Hemadri, Tosh, Sanyal, & Bandyopadhyay, 2005; Jamal & Belsham, 2015; Knowles et al., 2016; Reid et al., 2014). The transfer of these FMDV‐typing rRT‐PCR assays onto POCT platforms could further strengthen the diagnostic capacity of LMIC laboratories by offering a simple solution for rapid and improved FMDV characterization.

This study evaluates the performance of a commercially available lyophilized FMDV pan‐serotype‐specific rRT‐PCR assay (TC‐9092‐064; Tetracore, Inc., MD, USA), and a newly available East Africa‐specific typing assay (Bachanek‐Bankowska et al., 2016), both performed on the T‐COR™ 8 (Tetracore, Inc.), a portable, battery‐powered thermocycler (Almassian, Cockrell, & Nelson, 2013). The assessment was performed within laboratory and East African field settings, providing an approach that can type FMDV in situ using molecular methods.

## 2 MATERIALS AND METHODS

**Figure 1:**
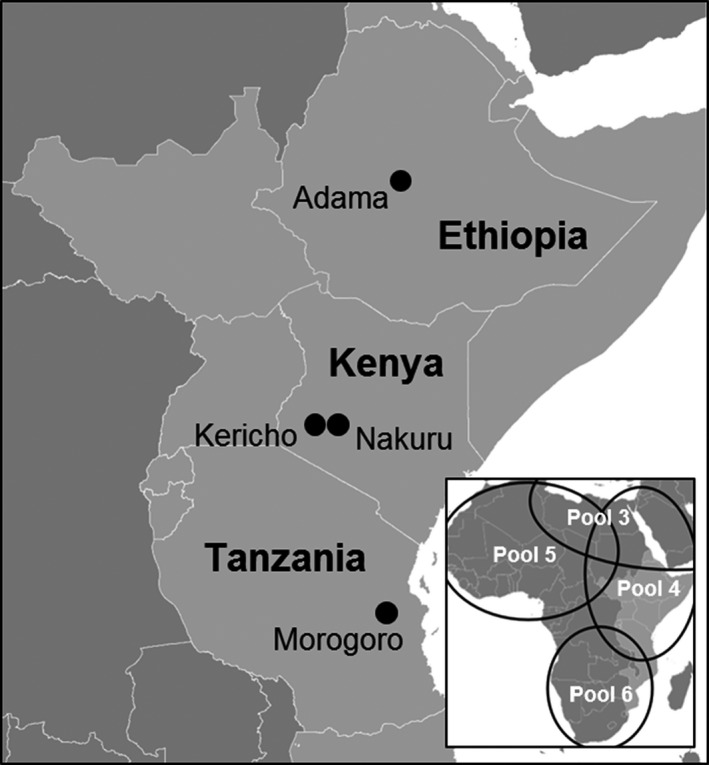
Field locations in East Africa. The bottom right map depicts the location of the antigenically defined foot‐and‐mouth disease virus pools within Africa (Paton et al., 2009). Field sampling locations are representative of foot‐and‐mouth disease virus pool 4

Laboratory work was carried out at The Pirbright Institute (UK), the Foot‐and‐Mouth Disease Laboratory (Embakasi, Kenya) and Sokoine University of Agriculture (SUA) (Morogoro, Tanzania). Field work was carried out in FMD‐endemic settings: Morogoro Region (Tanzania), Nakuru and Kericho County (Kenya) and Adama (Ethiopia) (Figure 1). These sites were selected on the basis of existing collaborations with investigators working on the epidemiology of FMD.

### 2.1 Ethics

Clinical samples used in this study for laboratory evaluation were archival samples generated in previous in vivo studies approved by The Pirbright Institute ethical review committee under the Animal Scientific Procedures Act (ASPA) and clinical field samples previously submitted to the World Reference Laboratory for FMD (WRLFMD; The Pirbright Institute, UK).

Field sampling in Tanzania was conducted as part of an ongoing research project under the Wellcome Trust Intermediate Fellowship FMD (WT104017MA, awarded to CJK), which aligned with the standards set in ASPA guidelines. Sampling in Kenya was carried out as part of a training programme run by The European Commission for the Control of Foot‐and‐Mouth Disease (EuFMD), and in Ethiopia, field sampling was carried out as part of an OIE twinning project “Strengthening the capacity of foot‐and‐mouth diagnosis and surveillance in Ethiopia and East Africa”.

### 2.2 Viruses and clinical samples for laboratory evaluation

#### 2.2.1 Sensitivity and specificity of lyophilized pan‐serotype‐specific rRT‐PCR reagents

Analytical sensitivity (limit of detection [LOD]) was determined using FMDV RNA extracted from decimal dilution series (10^−1^ to 10^−8^) of cell culture isolates O/TAN/39/2012 (topotype East Africa‐2); A/TAN/6/2013 (topotype AFRICA, lineage G‐I); SAT 1/KEN/72/2010 (topotype I [North West Zimbabwe]); SAT 2/KEN/2/2008 (topotype IV) diluted in negative bovine epithelial suspensions (10% [w/v] in M25 phosphate buffer: 35 mM Na_2_HPO_4_ , 5.7 mM KH_2_PO_4_, pH 7.6). RNA extraction was performed using the MagMAX™‐96 Viral RNA Isolation Kit (Applied Biosystems^®^, Thermo Fisher Scientific, MA, USA) following an automated nucleic acid extraction procedure on a KingFisher™ Flex (Thermo Fisher Scientific).

Diagnostic sensitivity was assessed using RNA extracted from a panel of 57 clinical samples (previously submitted to the WRLFMD), representing five serotypes (O, A, SAT 1, SAT 2 and Asia 1) from ten countries (Appendix S1). Diagnostic specificity was determined using RNA extracted from original epithelial suspensions from the following vesicular disease viruses: swine vesicular disease virus (UKG/24/1972; UKG/50/1972; UKG/51/1972; UKG/68/1972), vesicular stomatitis Indiana virus (VSIND1V) and vesicular stomatitis New Jersey virus (VSNJV).

The diagnostic window for detection (time points post‐infection when FMDV can be detected within clinical samples) was determined using RNA extracted from archival samples obtained from unvaccinated intradermolingual needle inoculated cattle, challenged with FMDV isolate A/TAI/17/2016 (topotype ASIA, lineage Sea‐97). Samples comprised of serum (*n* = 11) and mouth swabs (*n* = 11) taken daily from two animals from the day of challenge and epithelium (*n* = 4) and OP fluid (*n* = 2) collected from the same animals post‐mortem (carried out on the day of culling).

#### 2.2.2 Initial laboratory analysis of the lyophilized East Africa typing rRT‐PCR reagents

Initial laboratory analysis of the East Africa‐specific typing assay was performed on RNA extracted from 36 samples (serotypes O, A, SAT 1 and SAT 2) originating from East Africa (samples from Tanzania and Kenya included in Appendix S1). These samples were characterized by the WRLFMD using an antigen‐detection ELISA (Ferris & Dawson, 1988) and sequencing of the VP1 region (Knowles et al., 2016), as stated in the guidelines of the diagnostic manuals of the OIE (OIE, 2012).

#### 2.2.3 Determination of simple sample preparation protocols

Optimization of field‐ready protocols for the simple preparation of clinical samples prior to rRT‐PCR was carried out by preparing decimal dilution series (10^−1^ to 10^−9^) of FMDV isolate O/UAE/2/2003 (topotype Middle East‐South Asia, lineage Irn‐2001) in three separate diluents: negative bovine epithelium suspension, negative bovine serum and negative bovine OP fluid obtained from either archival experimental studies, or a UK abattoir post‐mortem. Aliquots were stored at −80°C until use. Nine sample preparation procedures were evaluated, based on their applicability to field settings (Table 1). The MagMAX™‐96 Viral RNA Isolation Kit/KingFisher™ Flex was used as the reference sample preparation procedure. Direct detection protocols were then tested on the unvaccinated needle inoculated cattle samples, described above.

**Table 1:** Details of sample preparation methods

Method	Procedure
MagMax™ (reference)	RNA was extracted using the MagMAX™ ‐ 96 Viral RNA Isolation Kit according to manufacturer guidelines (50 μl sample: 130 μl of lysis/binding solution, eluted in 90 μl; manual 1836M, revision H [Applied Biosystems^®^]) using a KingFisher™ Flex robot (Thermo Fisher Scientific)
MagMax™ (manual)	RNA was extracted as above using a DynaMag™‐ Spin magnet (Thermo Fisher Scientific). To meet biosecurity procedures the sample was added to lysis buffer before the magnetic beads
QIAamp^®^	QIAamp^®^ Viral RNA Mini Kit (Qiagen, UK) was used according to manufacturer's guidelines. RNA was extracted from 140 μl of sample (using the spin column protocol) and eluted in a final volume of 60 μl
1 in 5 dilution	Samples diluted 1 in 5 in NFW
1 in 10 dilution	Samples diluted 1 in 10 in NFW
1 in 20 dilution	Samples diluted 1 in 20 in NFW
Filter	Samples were diluted 1 in 5 in NFW and 1 ml of sample passed through an Acrodisc^®^ 25 mm syringe filter (w/0.1 μm Supor^®^ Membrane) (Pall Life Sciences, MI, USA)
Chelex^®^ 100	50 μl of 50% (w/v) Chelex^®^ 100 (Bio‐Rad, CA, USA) was added to 500 μl of pre‐diluted sample (1 in 5). Samples were vortexed, allowed to settle and the supernatant used in assays
Chelex^®^ (heat)	Samples were heated at 56°C for 10 min prior to processing with Chelex^®^ 100 as above
Ag‐LFD^a^	200 μl of sample was added to SVANODIP^®^ FMDV‐Ag LFDs (Boehringer Ingelheim, Bracknell, UK) and incubated at room temperature (25°C) for 72 hr. Nucleic acid was extracted from the loading pad and wicking strip of the Ag‐LFDs as previously published (Fowler et al., 2014)

NFW, nuclease free water; Ag‐LFD, antigen‐detection lateral flow device; FMDV, foot‐and‐mouth disease virus.

a Epithelium only.

Methods used for determination of simple sample preparation protocols in the laboratory.

### 2.3 Clinical samples for field evaluation

Samples (*n* = 144) from 78 individual cattle at 13 farms across East Africa were analysed in situ. Samples were collected opportunistically from locations where a local animal health worker or farmer reported the presence of clinical signs consistent with FMDV infection. This comprised of 13 cattle from two small holdings in Kericho County (Kenya, June 2016), 16 cattle from two small holdings in Nakuru County (Kenya, June 2016), 43 cattle from seven small holdings in Morogoro Rural and Mvomero Districts (Morogoro Region, Tanzania, September 2016) and six cattle from two small holdings in Adama (Ethiopia, October, 2016) (Figure 1). Samples were collected from cattle at different stages of infection (acute, convalescent and recovered) and included one or more of the following sample types per animal: serum, lesion swab, OP fluid and mouth/foot epithelium (Appendix S2). Cattle from the same herds that had no history or clinical signs of FMD at the time of the visit were also sampled (*n* = 12).

### 2.4 Sample preparation for field evaluation

Loose epithelial tissue of ruptured vesicular lesions from either the mouth or feet was prepared using the SVANODIP^®^ FMDV‐Ag Extraction Kit and SVANODIP^®^ FMDV‐Ag LFD Kit (Boehringer Ingelheim) as previously described (Howson et al., 2015). Epithelial samples from the feet (inter‐digital space/coronary band) were briefly washed in nuclease free water (NFW) prior to processing to remove soil contaminants. The homogenate was left to settle for 1 min, and then, the supernatant was removed and diluted one in 10 in NFW prior to analysis.

Swabs (GenoTube Livestock, Thermo Fisher Scientific) were collected by swabbing the surface of ruptured lesions in the mouth or on the feet (inter‐digital space/coronary band). The feet were cleaned in sterile water prior to swabbing in order to remove soil contaminants. Swabs were then agitated by hand in 1 ml NFW, which was used directly in analysis.

OP fluid was collected using a suitably sized probang cup following the guidelines within the diagnostic manuals of the OIE (2012) and diluted one in 10 in NFW prior to analysis.

Blood (10 ml) was collected from either the jugular or tail vein using Vacutainer^®^ Plus Plastic Serum Tubes (BD, Plymouth, UK), or similar. Samples were then transported back to the local laboratory and centrifuged at 3,000 *g* for 10 min. Serum was removed and then diluted one in 10 in NFW prior to analysis.

### 2.5 Real‐time RT‐PCR (rRT‐PCR)

#### 2.5.1 Laboratory‐based rRT‐PCR

An OIE‐recommended pan‐serotype‐specific one‐step rRT‐PCR was used as the reference assay. Each sample was tested in duplicate, using primers and probes reported by Callahan et al. (2002) and reagents, parameters and thermal cycling conditions as per Shaw et al. (2007). Positive reactions were defined as those which gave a detectable *C*_T_.

At The Pirbright Institute, rRT‐PCR was performed on a bench top real‐time PCR machine (Stratagene Mx3005p: Agilent Technologies, CA, USA) using nucleic acid extracted with the MagMAX™‐96 Viral RNA Isolation Kit and KingFisher™ Flex robot (as above). In East African laboratories, RNA was extracted using the QIAamp^®^ Viral RNA Mini Kit (Qiagen, UK) and rRT‐PCR reactions performed on either a PikoReal™ Real‐Time PCR System (Thermo Fisher Scientific) (Foot‐and‐Mouth Disease Laboratory, Kenya) or Applied Biosystems^®^ 7500 fast thermal cycler (Applied Biosystems^®^) (SUA, Tanzania).

#### 2.5.2 rRT‐PCR using the T‐COR™ 8

The FMDV lyophilized pan‐serotype‐specific assay was performed in duplicate using FMDV 2.0 reagents with inhibition control (TC‐9092‐064, Tetracore Inc.). This assay contains proprietary primers and probes to target two areas within the highly conserved 3D region of the FMDV genome (probes labelled with 6‐fluorescein amidite [FAM]), in addition to an exogenous internal control with corresponding primers and probes (probe labelled with Cy^®^ 5). The East Africa‐specific typing rRT‐PCR used the same chemistry, with the primers and probes as previously published in Bachanek‐Bankowska et al. (2016). In the typing assay, probes for each serotype were modified for multiplex detection using the following fluorescence channels: O (Dragonfly Orange™), A (FAM), SAT 1 (Cy^®^5) and SAT 2 (Texas Red^®^). Positive reactions were defined as those which gave a detectable *C*_T_.

To each reaction, 20 μl of resuspension buffer and 5 μl of sample were added. Reactions were performed on a T‐COR™ 8 (Tetracore, Inc.) with the following cycling conditions: 48°C for 15 min, 95°C for 2 min, followed by 45 cycles of 95°C for 10 s and 60°C for 40 s.

### 2.6 Biosafety procedures in the field

During field work, a biosafety boundary was established outside of each farm premises, to separate livestock‐containing areas (considered contaminated) from livestock‐free areas (considered uncontaminated). This was established to ensure that field work did not contribute to the further spread of FMDV. Sample preparation and assay assembly was performed inside the contaminated area. Assembled reactions (rRT‐PCR master mix plus sample) were surface disinfected with either citric acid (0.2% w/v) or FAM^®^ 30 (1:240) prior to transfer to the uncontaminated area where rRT‐PCR on the T‐COR™ 8 was performed. Different personnel were present in these areas to facilitate the transfer of samples. Personnel entering the contaminated area donned disposable over‐suits, two pairs of gloves and over‐shoes which were surface disinfected as above prior to disposal within a bag (along with disposable consumables) for incineration at local laboratory facilities. Any non‐disposable equipment which was used in the contaminated area (e.g., probang cup, forceps, scissors and boots) was suitably surface disinfected prior to transfer back to the uncontaminated area. Following each test and between farms, the T‐COR™ 8 was surface disinfected (as above) and sprayed with DNAZap™ PCR DNA Degradation Solutions (Thermo Fisher Scientific). At the end of field work, the T‐COR™ 8 and non‐disposable equipment were surface disinfected (as above) prior to fumigation with formaldehyde (10 mg/m^3^) at 30°C (relative humidity > 70 for > 60 min). Samples for concordance testing were added to MagMAX™‐96 Viral RNA lysis buffer within the contaminated area, surface disinfected (as above) for transfer to the uncontaminated area and transported (double‐contained) to appropriate local laboratory facilities on ice.

### 2.7 Statistical analysis

Cohen's Kappa statistic (κ) and the proportion of observed agreement (*A*_obs_) were used to measure the agreement between diagnostic tests. Statistical analyses were performed using R (R Core Team, 2014).

## 3 RESULTS

### 3.1 Sensitivity and specificity of lyophilized pan‐serotype‐specific rRT‐PCR reagents

**Figure 2:**
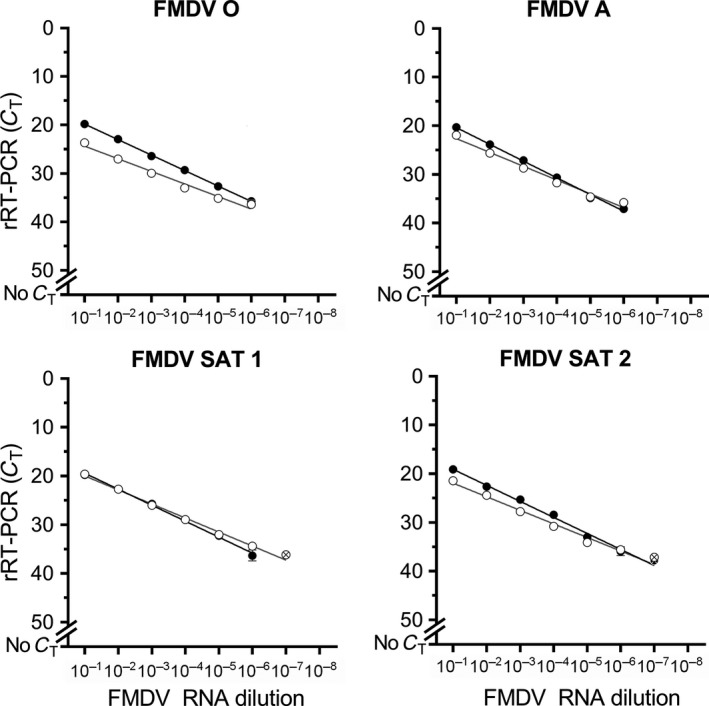
Limit of detection analysis for lyophilized pan‐serotype‐specific reagents compared to the reference real‐time reverse transcription PCR (rRT‐PCR) across four different serotypes (O/TAN/39/2012; A/TAN/6/2013; Southern African Territories [SAT] 1/KEN/72/2010; SAT 2/KEN/2/2008). (●) reference rRT‐PCR performed on a benchtop thermocycler; (○) pan‐serotype‐specific lyophilized reagents performed on the T‐COR™ 8. Points represent the mean of two replicates; crossed points indicate that of the identical replicates, one was positive and the other negative. The error bars indicate the range

**Figure 3:**
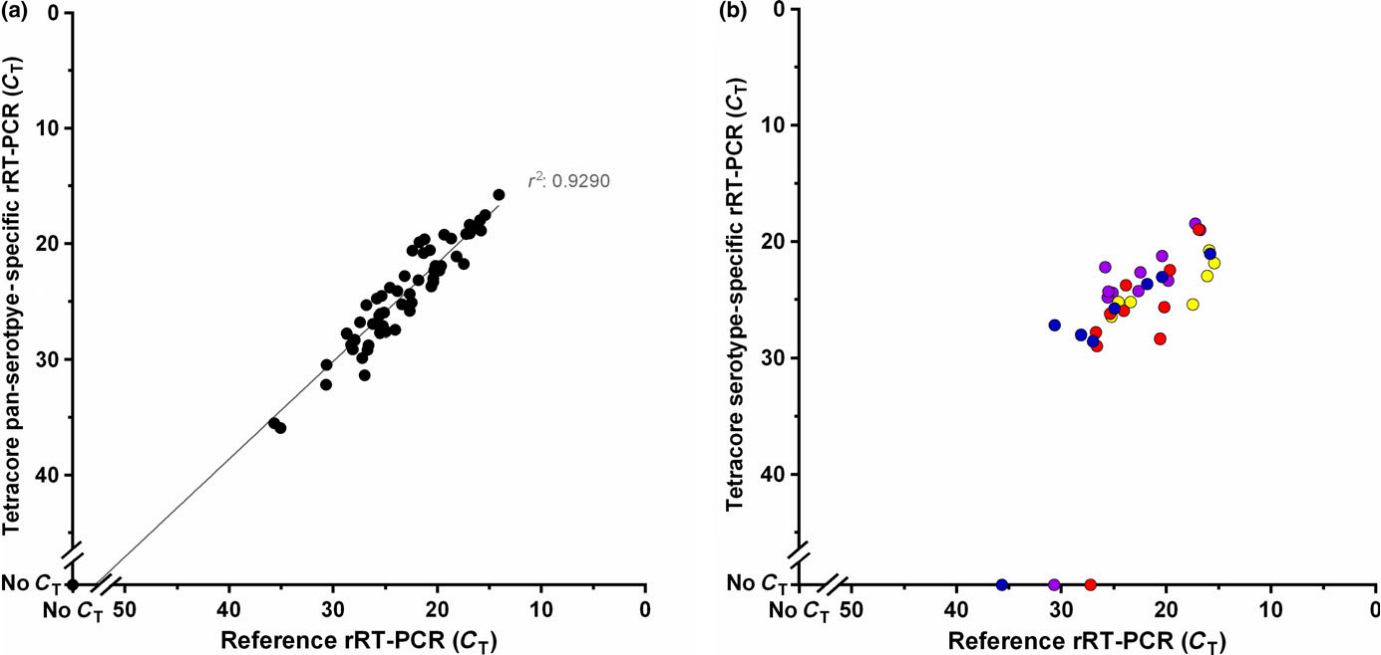
Comparison between lyophilized pan‐serotype‐specific reagents and reference real‐time reverse transcription PCR. (a) Pan‐serotype‐specific rRT‐PCR compared to the reference rRT‐PCR across a panel of 57 foot‐and‐mouth disease virus (FMDV)‐positive clinical samples. (b) Serotype‐specific rRT‐PCR compared to the reference rRT‐PCR across a panel of 36 FMDV‐positive clinical samples from East Africa. The colour of points indicates serotype: blue (A), red (O), yellow (Southern African Territories [SAT] 1) and purple (SAT 2). For both graphs, points represent the mean of two replicates

**Figure 4:**
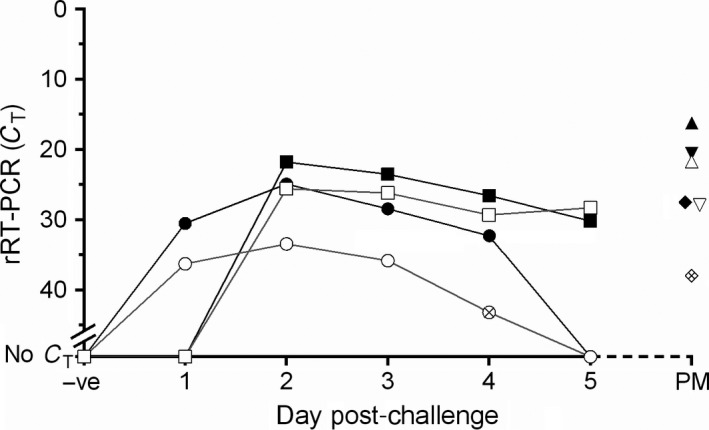
Determination of clinical detection window for the pan‐serotype‐specific lyophilized real‐time reverse transcription PCR reagents. Assays performed on extracted RNA on the T‐COR™ 8 included the following sample types: (●) serum; (■) mouth swabs; (▲) foot epithelium; (▼) mouth epithelium; (♦) oesophageal–pharyngeal (OP) fluid. Assays performed directly (one in 10 dilution) on samples using the T‐COR™ 8 included the following sample types: (○) serum; (□) mouth swabs; (▵) foot epithelium; (▽) mouth epithelium; (♢) OP fluid. Points represent the mean of two replicates and crossed points represent when one replicate was positive and the other negative

The analytical sensitivity of the lyophilized pan‐serotype‐specific reagents, performed on the T‐COR™ 8, was equivalent to the reference rRT‐PCR across the four serotypes tested (both consistently detected down to 10^−6^ for each serotype) (Figure 2). Diagnostic sensitivity of the pan‐serotype‐specific lyophilized assay was 100% across the 57 FMDV‐positive samples tested (Appendix S1), with all diagnostic samples tested displaying comparable *C*_T_ values to the reference rRT‐PCR (Figure 3a). The specificity of the pan‐serotype‐specific lyophilized assay was also 100% (*n* = 6), in that no cross‐reactivity was observed with the other vesicular disease viruses tested (Appendix S1). FMDV RNA was detected in serum from 1–4 days post‐challenge, mouth swabs from 2 days onwards post‐challenge and all epithelium and OP samples collected post‐mortem using the lyophilized pan‐serotype‐specific reagents (Figure 4).

### 3.2 Initial laboratory analysis of the lyophilized East Africa‐specific typing rRT‐PCR reagents

For the East African clinical samples tested in the laboratory (*n* = 36), the serotype‐specific typing assay detected 7/8 serotype A, 9/10 serotype O, 7/7 serotype SAT 1 and 10/11 serotype SAT 2 (Appendix S1 and Figure 3b). The three samples for which no serotype was detected generated high *C*_T_ values on the reference rRT‐PCR (values of 35.64, 27.18 and 30.65). No cross‐reactivity among serotypes was observed for any of the clinical samples tested.

### 3.3 Determination of simple sample preparation protocols

**Figure 5:**
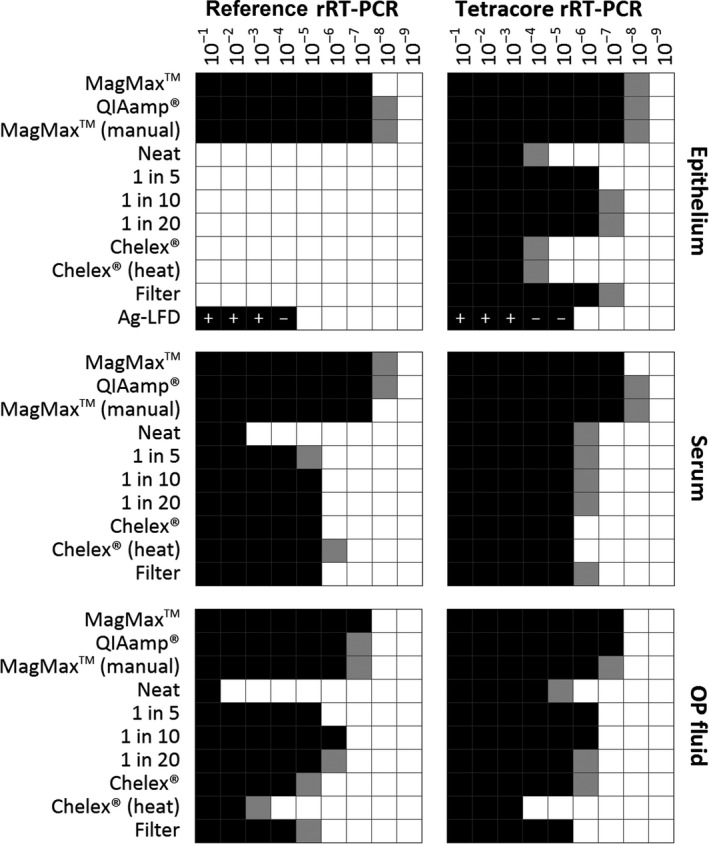
Comparison of sample preparation methods for the reference real‐time reverse transcription PCR (rRT‐PCR) and lyophilized pan‐serotype‐specific rRT‐PCR. Sample preparation methods were tested for three sample types (epithelial suspensions, sera and oesophageal–pharyngeal [OP] fluid) across a dilution series (10^−1^ to 10^−9^). Elution from antigen‐detection lateral‐flow devices (Ag‐LFD) was tested for epithelial suspensions only: ± represent Ag‐LFD results. Black squares represent dilutions where both replicates were positive for FMDV; grey squares represent dilutions where one replicate was positive; white squares represent reactions where both replicates were negative for FMDV. For the reference rRT‐PCR, the use of simple sample preparation methods for epithelium resulted in assay inhibition in known positive samples (bar the use of Ag‐LFD)

The use of simple extraction kits (QIAmp^®^ and MagMax™ [manual]) achieved similar analytical sensitivity across all three sample types tested in comparison with the reference sample preparation procedure (MagMax™ extraction) for both the reference rRT‐PCR and lyophilized rRT‐PCR (Figure 5). Simple sample preparation protocols of epithelial material resulted in complete inhibition of the reference rRT‐PCR (Figure 5). However, when these same sample preparations were tested with the lyophilized rRT‐PCR reagents (e.g., dilution in NFW and the use of a filter), a log_10_ reduction in analytical sensitivity compared with the use of extracted RNA. Alternative methods, such as the use of use of Chelex^®^ 100 or elution from Ag‐LFDs, did not increase analytical sensitivity for either rRT‐PCR assay (Figure 5).

Although serum could be added directly to both the reference rRT‐PCR and lyophilized rRT‐PCR, the analytical sensitivity was higher with the lyophilized rRT‐PCR. For all other methods tested, the analytical sensitivity for the two rRT‐PCR assays was similar, with dilution in NFW and/or the use of Chelex^®^ 100 or a syringe filter required to improve the LOD (Figure 5). For OP fluid, a log_10_ reduction (relative to MagMax™ extraction) was evident for both rRT‐PCR reagents when diluted one in 10 in NFW prior to analysis. The additional use of syringe filters, Chelex^®^ 100 or other dilutions resulted in a further decrease in LOD (Figure 5).

Dilutions of one in 10 of un‐extracted clinical samples (animal study samples) enabled FMDV RNA to be detected in 26/28 FMDV‐positive samples (defined using the reference rRT‐PCR with extracted RNA) using the lyophilized pan‐serotype‐specific rRT‐PCR (Figure 4), comparatively to 19/28 for the reference rRT‐PCR (data not shown). The two discordant samples (false‐negatives) for the lyophilized pan‐serotype‐specific rRT‐PCR had *C*_T_ values of 35.3 (serum 4 days post‐challenge) and 29.0 (OP fluid taken post‐mortem) when using extracted RNA.

### 3.4 Detection of FMDV in situ using lyophilized rRT‐PCR in East Africa

The T‐COR™ 8 and the newly developed rRT‐PCR protocols were tested on 144 samples from 78 cattle across 13 farms in East Africa.

**Figure 6:**
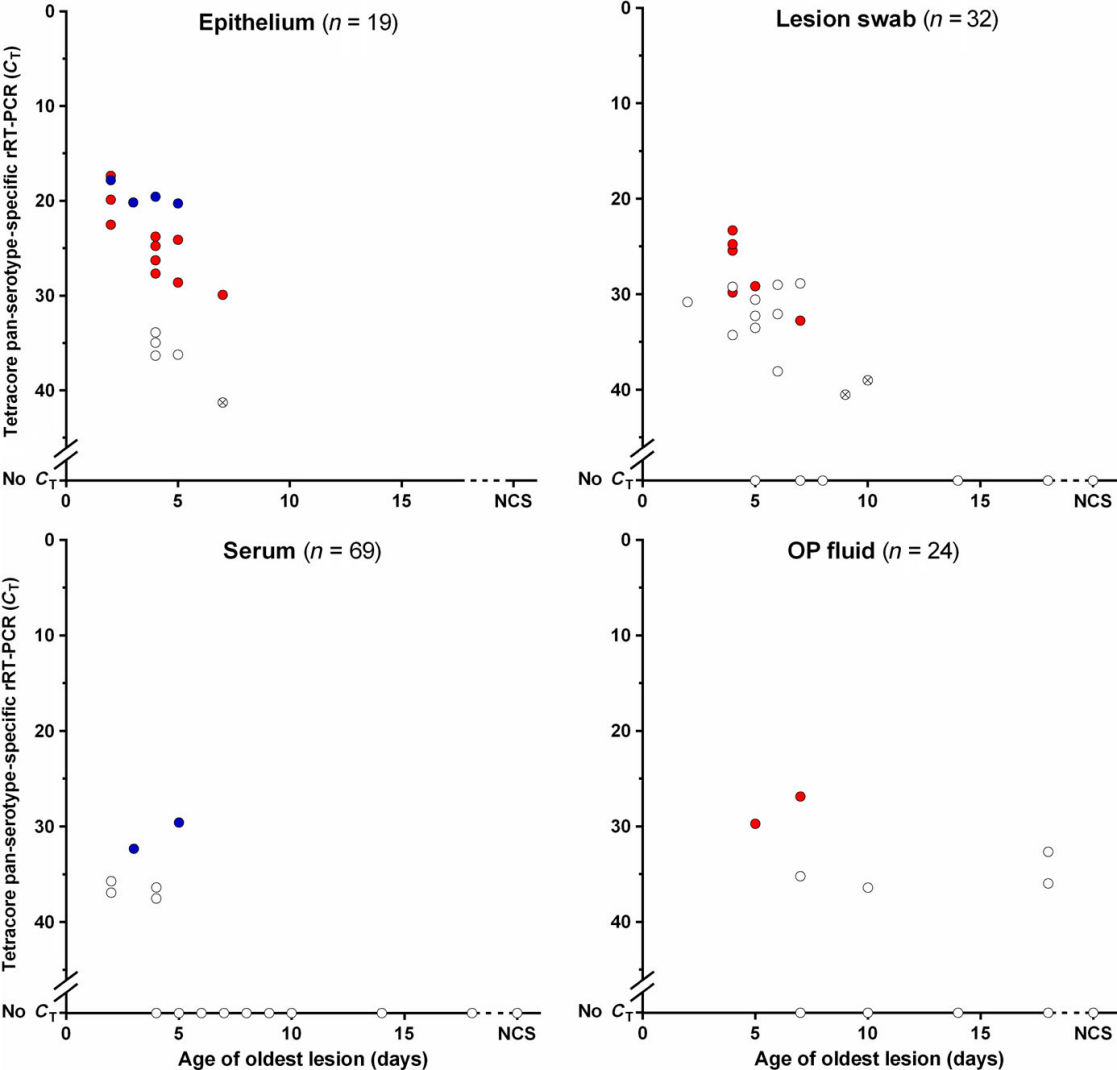
In situ real‐time reverse transcription PCR (rRT‐PCR) results for 144 East African samples using lyophilized rRT‐PCR reagents and the T‐COR™ 8. Samples were collected from cattle displaying clinical signs of foot‐and‐mouth disease and cattle with no clinical signs (NCS). Each point represents an average for a single sample (tested in duplicate) on the pan‐serotype‐specific rRT‐PCR (samples that share the same *C*_T_ will only appear as a single point with individual *C*_T_ values presented within supplementary data); crossed points indicate that out of the duplicates, one was positive and the other negative. Positive samples were then tested using the typing assay; the colour of the points represents the serotype detected: blue (serotype A), red (serotype O) and white (serotype not detected). The 22/46 FMDV‐positive samples where the serotype was not detected had pan‐serotype‐specific *C*_T_ values of > 29 (using lyophilized reagents)

Using the pan‐serotype‐specific lyophilized rRT‐PCR in combination with the T‐COR™ 8, FMDV RNA was identified in 5/5 epithelial, 1/1 swab and 3/3 sera samples collected from cattle displaying 1‐ to 3‐day‐old lesions. FMDV RNA was identified in 13/14 epithelial, 15/19 swab, 3/4 OP fluid and 3/29 sera samples collected from cattle displaying 4‐ to 7‐day‐old lesions. Of the 27 cattle that displayed lesions older than 1 week (8+ days post‐initial lesion presentation), FMDV RNA was identified in 3/12 OP fluid samples, whilst all sera (*n* = 25) and swab (*n* = 10) samples were negative. FMDV was not detected in any OP fluid (*n* = 8), mouth swab (*n* = 2) or sera (*n* = 12) sample collected from the 12 clinically negative cattle (Figure 6) (Appendix S2).

Samples considered positive by the lyophilized pan‐serotype‐specific assay (*n* = 46), in addition to a selection of samples from cattle considered clinically negative (*n* = 7), were then subsequently characterized using the East Africa‐specific typing assay which has been developed to detect all known FMDV strains relevant to this region (Bachanek‐Bankowska et al., 2016). Of these, 24 were identified as either A or O (Figure 6); no amplification was present for samples collected from clinically FMD‐negative cattle. FMDV‐positive samples where the serotype was not detected (*n* = 22) had pan‐serotype‐specific *C*_T_ values of > 29 (using lyophilized reagents) (Appendix S2).

**Figure 7:**
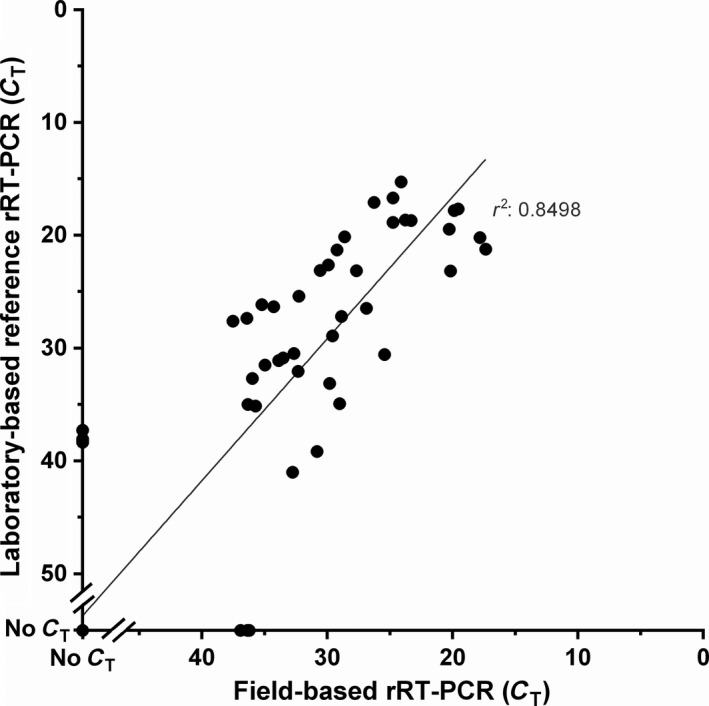
Comparison between real‐time reverse transcription PCR (rRT‐PCR) results performed using lyophilised pan‐serotype specific rRT‐PCR in the field and reference rRT‐PCR performed in local laboratories

For comparison, 99 of the samples tested on the pan‐serotype‐specific lyophilized rRT‐PCR (using the T‐COR™ 8) were also tested using the reference rRT‐PCR within local laboratory settings in East Africa. High agreement was apparent (κ = 0.864, *p* = 2.109e‐15, *A*_obs_^ ^= 0.936) between pan‐serotype‐specific assays in terms of positive and negative test results (Figure 7), with all discordant results between tests having *C*_T_ values of >36 (Appendix S2), which is at the threshold of the analytical sensitivity of both tests.

## 4 DISCUSSION

The requirement for rapid diagnosis of FMD has led to an increase in the development and evaluation of POCT for FMDV detection. In addition to providing a means to rapidly confirm cases of FMD on or close to the farm during outbreaks, these technologies could potentially provide a route for LMICs to establish robust field/laboratory testing capacity. For example, immunological‐based assays such as lateral‐flow devices (Ag‐LFDs) have been developed for rapid viral antigen detection and successfully tested in situ during the UK 2007 FMDV outbreak (Ryan et al., 2008). However, Ag‐LFDs have only been validated for use with epithelial suspension and vesicular fluid, and limited analytical sensitivity restricts their usefulness to the acute stage of infection (Ferris et al., 2009, 2010). As a consequence, attempts to transfer highly sensitive rRT‐PCR assays onto portable detection platforms have been investigated (Hearps et al., 2002; Howson et al., 2015; Madi et al., 2012; Monwina et al., 2007; Paixão et al., 2008), but are either not commercially available or are only suitable for research purposes (i.e., not diagnostic use). This study evaluates the performance of a commercially available, lyophilized FMDV pan‐serotype‐specific assay, in combination with a commercially available portable thermocycler, in laboratory and East African field settings. Such tests have the potential to contribute valuable epidemiological information to support country‐level and regional control programmes, such as the Progressive Control Pathway for FMD (Sumption et al., 2012). The future success and implementation of such technologies depends upon several factors, including dissemination of information and adoption of these methodologies into current diagnostic strategies.

The provision of reagents in a lyophilized kit format simplifies reagent storage by negating the requirement for a cold chain, whilst minimizing the requirement for skilled personnel and multiple pipetting stages by streamlining assay set up (assays only require addition of a resuspension buffer and sample). The lyophilized pan‐serotype‐specific reagents, in combination with the T‐COR™ 8, maintained comparable diagnostic performance to the reference rRT‐PCR when evaluated with extracted RNA. A one to two log_10_ reduction in analytical sensitivity for the lyophilized pan‐serotype‐specific reagents was evident when no extraction method was used; however, performance was still equivalent to the sensitivity of the reference rRT‐PCR when a diagnostic cut‐off of *C*_T_ <32 (as recommended by Shaw et al., 2007) was applied to the reference rRT‐PCR. As the lyophilized pan‐serotype‐specific reagents are still able to perform rRT‐PCR in the absence of extraction, they offer a potential solution for molecular POCT in field settings, which is not possible using the reference rRT‐PCR.

Throughout field validation of the T‐COR™ 8 in East Africa, lyophilized reagents generated results consistent with clinical observations, with FMDV detected in samples from the early onset of infection through to delayed viral clearance. Results were gained in less than 1.5 hr from sample collection. High concordance was evident between results gained in the field (T‐COR™ 8 rRT‐PCR) and local East African laboratories (reference rRT‐PCR), with any disagreements associated with samples that had *C*_T_ values above the diagnostic threshold of *C*_T_ < 32 (Shaw et al., 2007). Although the provision of simple FMDV‐positive/negative results is sufficient for the confirmation of FMD during outbreaks, the value of this information is limited in countries where FMD is endemic. In these situations, it is beneficial to characterize circulating FMDV outbreaks in order to make tailored control programmes a realistic possibility (Namatovu et al., 2013). In support of this, this study also evaluated a lyophilized version of a published FMDV‐typing assay specific to East Africa (Bachanek‐Bankowska et al., 2016). The typing assay was able to characterize FMDV present in four different sample types collected from seven small holdings (three in Kenya, three in Tanzania and one in Ethiopia) where cattle were presenting two to seven‐day‐old FMD lesions. Samples which were unable to be typed (*n* = 22) were all considered as weak positives by the lyophilized pan‐serotype‐specific rRT‐PCR (*C*_T_ > 29) and therefore at the threshold at which characterization information can be routinely obtained by sequencing. The typing assay performance in field settings is therefore consistent with the ability to obtain characterization data within laboratory settings. Two serotypes were detected during the period of testing (O and A), leading to the first reported characterization of FMDV at the pen‐side using molecular methods.

In conclusion, this publication describes the laboratory and field evaluation of lyophilized FMDV‐specific rRT‐PCR assays in combination with a portable thermocycler. The simplicity of the T‐COR™ 8 to operate, combined with robust lyophilized reagents, high sensitivity and compatibility with simple sample preparation methods, demonstrates an important transition for FMDV‐specific rRT‐PCR assays into formats suitable for use on or close to the point‐of‐care.
