# Supplementary material for: Direct detection and characterization of foot‐and‐mouth disease virus in East Africa using a field‐ready real‐time PCR platform
Source: Transbound Emerg Dis. 2017 Jul 30;65(1):221–31. doi: 10.1111/tbed.12684 (PMC5811823; doi:10.1111/tbed.12684)
Supplement: Supplementary file 1 [file TBED-65-221-s001.docx]

**Appendix S1.** FMDV clinical samples used to determine diagnostic sensitivity and specificity of assays in laboratory settings

| Serotype | WRLFMD sample ID | Topotype | Lineage | Location | Year | Type* | Reference  rRT-PCR C_T_** | Lyophilised rRT-PCR C_T_** | Typing  rRT-PCR C_T_** |
| --- | --- | --- | --- | --- | --- | --- | --- | --- | --- |
| O | HKN/12/2015 | CATHAY | unnamed | Hong Kong | 2015 | OS | 28.26 | 28.70 | N/A |
|  | IRN/26/2015 | ME-SA | PanAsia-2^BAL-09^ | Iran | 2015 | OS | 28.67 | 27.75 | N/A |
|  | KUW/1/2016 | ME-SA | PanAsia-2^BAL-09^ | Kuwait | 2016 | OS | 16.86 | 18.35 | N/A |
|  | KUW/4/2016 | ME-SA | PanAsia-2^BAL-09^ | Kuwait | 2016 | OS | 14.06 | 15.75 | N/A |
|  | PAK/30/2015 | ME-SA | PanAsia-2^BAL-09^ | Pakistan | 2015 | OS | 21.20 | 19.60 | N/A |
|  | PAK/32/2015 | ME-SA | PanAsia-2^BAL-09^ | Pakistan | 2015 | OS | 21.30 | 20.80 | N/A |
|  | PAK/34/2015 | ME-SA | PanAsia-2^BAL-09^ | Pakistan | 2015 | OS | 20.70 | 20.55 | N/A |
|  | PAT/4/2015 | ME-SA | PanAsia | Palestine | 2015 | OS | 27.90 | 28.30 | N/A |
|  | TAN/5/2009 | EA-2 | - | Tanzania | 2012 | OS | 23.82 | 24.10 | 23.75 |
|  | TAN/39/2012 | EA-2 | unnamed | Tanzania | 2012 | OS | 24.03 | 27.45 | 25.95 |
|  | TAN/3/2014 | EA-2 | unnamed | Tanzania | 2014 | OS | 26.68 | 29.15 | 27.80 |
|  | TAN/6/2014 | EA-2 | unnamed | Tanzania | 2014 | OS | 26.57 | 28.75 | 28.95 |
|  | KEN/10/2009 | EA-2 | - | Kenya | 2009 | OS | 27.18 | 29.85 | No C_T_ |
|  | KEN/146/2010 | EA-2 | unnamed | Kenya | 2010 | OS | 16.87 | 19.10 | 18.95 |
|  | KEN/151/2010 | EA-2 | unnamed | Kenya | 2010 | OS | 20.54 | 23.70 | 28.35 |
|  | KEN/152/2010 | EA-2 | unnamed | Kenya | 2010 | OS | 20.15 | 21.90 | 25.65 |
|  | KEN/154/2010 | EA-2 | unnamed | Kenya | 2010 | OS | 25.33 | 24.50 | 26.20 |
|  | KEN/15/2011 | EA-2 | unnamed | Kenya | 2011 | OS | 19.61 | 21.90 | 22.45 |
| A | IRN/21/2015 | ASIA | G-VII | Iran | 2015 | OS | 27.39 | 26.80 | N/A |
|  | IRN/24/2015 | ASIA | Iran-05^SIS-10^ | Iran | 2015 | OS | 21.68 | 19.85 | N/A |
|  | SAU/8/2015 | ASIA | G-VII | Saudi Arabia | 2015 | OS | 26.78 | 25.30 | N/A |
|  | PAK/31/2015 | ASIA | Iran-05^FAR-11^ | Pakistan | 2015 | OS | 18.63 | 19.55 | N/A |
|  | PAK/56/2015 | ASIA | Iran-05^FAR-11^ | Pakistan | 2015 | OS | 22.63 | 25.80 | N/A |
|  | TAN/42/2009 | AFRICA | G-I | Tanzania | 2009 | OS | 21.79 | 23.15 | 23.65 |
|  | TAN/56/2012 | AFRICA | G-I | Tanzania | 2012 | OS | 35.64 | 35.50 | No C_T_ |
|  | TAN/61/2012 | AFRICA | G-I | Tanzania | 2012 | OS | 26.96 | 31.35 | 28.55 |
|  | TAN/70/2012 | AFRICA | G-I | Tanzania | 2012 | OS | 20.36 | 23.00 | 23.05 |
|  | TAN/71/2012 | AFRICA | G-I | Tanzania | 2012 | OS | 24.91 | 27.60 | 25.75 |
|  | TAN/15/2013 | AFRICA | G-I | Tanzania | 2013 | OS | 15.78 | 18.85 | 21.05 |
|  | KEN/28/2008 | AFRICA | G-I | Kenya | 2008 | OS | 30.61 | 30.45 | 27.20 |
|  | KEN/22/2009 | AFRICA | G-I | Kenya | 2009 | OS | 28.09 | 29.10 | 28.00 |
| SAT 1 | TAN/50/2012 | I (NWZ) | unnamed | Tanzania | 2012 | OS | 25.21 | 27.10 | 26.45 |
|  | TAN/23/2013 | I (NWZ) | - | Tanzania | 2013 | OS | 15.87 | 17.95 | 20.75 |
|  | TAN/29/2013 | I (NWZ) | - | Tanzania | 2013 | OS | 15.38 | 17.50 | 21.85 |
|  | TAN/22/2014 | I (NWZ) | unnamed | Tanzania | 2014 | OS | 17.44 | 21.75 | 25.40 |
|  | KEN/26/2008 | I (NWZ) | - | Kenya | 2008 | OS | 16.05 | 18.35 | 22.95 |
|  | KEN/9/2009 | I (NWZ) | - | Kenya | 2009 | OS | 23.37 | 25.25 | 25.20 |
|  | KEN/12/2009 | I (NWZ) | - | Kenya | 2009 | OS | 24.54 | 23.80 | 25.20 |
| SAT 2 | ZIM/9/2015 | II | unnamed | Zimbabwe | 2015 | OS | 25.44 | 26.05 | N/A |
|  | ZIM/21/2015 | II | unnamed | Zimbabwe | 2015 | OS | 26.16 | 26.95 | N/A |
|  | TAN/3/2011 | IV | IV | Tanzania | 2011 | OS | 25.08 | 25.95 | 24.40 |
|  | TAN/7/2011 | IV | IV | Tanzania | 2011 | OS | 22.63 | 24.35 | 24.25 |
|  | TAN/5/2012 | IV | unnamed | Tanzania | 2012 | OS | 20.37 | 23.30 | 21.25 |
|  | TAN/10/2012 | IV | unnamed | Tanzania | 2012 | OS | 19.77 | 22.30 | 23.35 |
|  | TAN/14/2012 | IV | unnamed | Tanzania | 2012 | OS | 22.44 | 25.10 | 22.65 |
|  | TAN/19/2012 | IV | unnamed | Tanzania | 2012 | OS | 25.78 | 24.75 | 22.20 |
|  | TAN/64/2012 | IV | unnamed | Tanzania | 2012 | OS | 25.56 | 26.20 | 24.80 |
|  | KEN/2/2007 | IV | - | Kenya | 2007 | OS | 30.65 | 32.15 | No C_T_ |
|  | KEN/12/2011 | IV | unnamed | Kenya | 2011 | OS | 16.74 | 18.85 | 19.00 |
|  | KEN/21/2011 | IV | unnamed | Kenya | 2011 | OS | 17.19 | 19.15 | 18.45 |
|  | KEN/4/2012 | IV | unnamed | Kenya | 2012 | OS | 25.48 | 27.70 | 24.30 |
|  | SUD/7/2014 | VII | Alx-12 | Sudan | 2015 | OS | 35.03 | 35.90 | N/A |
| Asia 1 | IRN/20/2015 | ASIA | Sindh-08 | Iran | 2015 | OS | 22.37 | 20.60 | N/A |
|  | PAK/28/2015 | ASIA | Sindh-08 | Pakistan | 2015 | OF | 20.27 | 22.30 | N/A |
|  | PAK/29/2015 | ASIA | Sindh-08 | Pakistan | 2015 | OF | 18.13 | 21.1 | N/A |
|  | PAK/33/2015 | ASIA | Sindh-08 | Pakistan | 2015 | OS | 19.31 | 19.20 | N/A |
|  | PAK/43/2015 | ASIA | Sindh-08 | Pakistan | 2015 | OS | 23.11 | 22.80 | N/A |
| SVDV | UKG/24/1972 | Undefined | Undefined | UK | 1972 | CC | No C_T_ | No C_T_ | No C_T_ |
|  | UKG/50/1972 | Undefined | Undefined | UK | 1972 | CC | No C_T_ | No C_T_ | No C_T_ |
|  | UKG/51/1972 | Undefined | Undefined | UK | 1972 | CC | No C_T_ | No C_T_ | No C_T_ |
|  | UKG/68/1972 | Undefined | Undefined | UK | 1972 | CC | No C_T_ | No C_T_ | No C_T_ |
| VSIND1V | Colorado/1942 | Undefined | Undefined | USA | 1942 | CC | No C_T_ | No C_T_ | No C_T_ |
| VSNJV | Columbia/1964 | Undefined | Undefined | Colombia | 1964 | CC | No C_T_ | No C_T_ | No C_T_ |

*OS (original suspension); OF (original fluid e.g. vesicular fluid); CC (cell culture)

**C_T_ value is the average value across two replicates for real-time reverse transcription PCR (rRT-PCR)

WRLFMD: World Reference Laboratory for Foot-and-Mouth Disease; FMDV: foot-and-mouth disease virus; SAT: Southern African Territories, SVDV: swine vesicular disease virus VSIND1V: vesicular stomatitis Indiana virus; VSNJV: vesicular stomatitis New Jersey virus.
