# Supplementary material for: Direct detection and characterization of foot‐and‐mouth disease virus in East Africa using a field‐ready real‐time PCR platform
Source: Transbound Emerg Dis. 2017 Jul 30;65(1):221–31. doi: 10.1111/tbed.12684 (PMC5811823; doi:10.1111/tbed.12684)
Supplement: Supplementary file 2 [file TBED-65-221-s002.docx]

| **Appendix S2**: Clinical samples used to evaluate the performance of the T-COR^TM^ 8 in field settings | | | | | | | | | |
| --- | --- | --- | --- | --- | --- | --- | --- | --- | --- |
| Animal reference | Location | Lesion age  (approximate) | Sample | T-COR^TM^ 8  pan-serotype specific | T-COR^TM^ 8  serotype specific | | | | **Laboratory  pan-serotype specific  (reference) |
|  |  |  |  |  | FAM  (A) | DFO  (O) | Cy®5  (SAT 1) | TxR  (SAT 2) |  |
| Cow_1 | Kericho County, Kenya  (Farm 1) | 4 days | Serum | 36.40 | No C_T_ | No C_T_ | No C_T_ | No C_T_ | No C_T_ |
| Cow_2 |  | 7 days | Serum | No C_T_ | N/A | N/A | N/A | N/A | No C_T_ |
| Cow_3 |  | 10 days | Serum | No C_T_ | N/A | N/A | N/A | N/A | No C_T_ |
| Cow_4 |  | 6 days | Serum | No C_T_ | N/A | N/A | N/A | N/A | No C_T_ |
| Cow_5 |  | 10 days | Serum | No C_T_ | N/A | N/A | N/A | N/A | No C_T_ |
| Cow_6 |  | 10 days | Serum | No C_T_ | N/A | N/A | N/A | N/A | No C_T_ |
| Cow_7 |  | 7 days | Serum | No C_T_ | N/A | N/A | N/A | N/A | No C_T_ |
| Cow_8 |  | 10 days | Serum | No C_T_ | N/A | N/A | N/A | N/A | No C_T_ |
| Cow_9 | Kericho County, Kenya  (Farm 2) | 5 days | Epithelium | 20.25 | 21.00 | No C_T_ | No C_T_ | No C_T_ | 19.44 |
|  |  |  | Serum | 29.55 | 27.15 | No C_T_ | No C_T_ | No C_T_ | 28.88 |
| Cow_10 |  | 5 days | Serum | No C_T_ | N/A | N/A | N/A | N/A | No C_T_ |
| Cow_11 |  | 3 days | Epithelium | 20.15 | 22.20 | No C_T_ | No C_T_ | No C_T_ | 23.14 |
|  |  |  | Serum | 32.30 | 30.50 | No C_T_ | No C_T_ | No C_T_ | 32.02 |
| Cow_12 |  | 2 days | Epithelium | 17.80 | 22.25 | No C_T_ | No C_T_ | No C_T_ | 20.17 |
| Cow_13 |  | 4 days | Epithelium | 19.55 | 21.70 | No C_T_ | No C_T_ | No C_T_ | 17.64 |
| Cow_14 | Nakuru County, Kenya  (Farm 3) | 4 days | Lesion swab | 25.40 | No C_T_ | 26.70 | No C_T_ | No C_T_ | 30.53 |
|  |  |  | Serum | No C_T_ | N/A | N/A | N/A | N/A | No C_T_ |
| Cow_15 |  | 4 days | Lesion swab | 29.80 | No C_T_ | 32.80 | No C_T_ | No C_T_ | 33.09 |
| Cow_16 |  | 7 days | Lesion swab | 32.75 | No C_T_ | 34.40 | No C_T_ | No C_T_ | 40.97 |
| Cow_17 |  | 10 days | Lesion swab | 39.00* | N/A | N/A | N/A | N/A | No C_T_ |
| Cow_18 | Nakuru County, Kenya  (Farm 4) | 7 days | Lesion swab | No C_T_ | N/A | N/A | N/A | N/A | No C_T_ |
|  |  |  | Serum | No C_T_ | N/A | N/A | N/A | N/A | No C_T_ |
| Cow_19 |  | 5 days | Epithelium | 36.2 | No C_T_ | No C_T_ | No C_T_ | No C_T_ | No C_T_ |
|  |  |  | Serum | No C_T_ | N/A | N/A | N/A | N/A | No C_T_ |
| Cow_20 |  | 8 days | Lesion swab | No C_T_ | N/A | N/A | N/A | N/A | No C_T_ |
|  |  |  | Serum | No C_T_ | N/A | N/A | N/A | N/A | No C_T_ |
| Cow_21 |  | 7 days | Lesion swab | 28.85 | No C_T_ | No C_T_ | No C_T_ | No C_T_ | 27.17 |
|  |  |  | Serum | No C_T_ | N/A | N/A | N/A | N/A | No C_T_ |
|  |  |  | OP fluid | 26.85 | No C_T_ | 25.25 | No C_T_ | No C_T_ | 26.45 |
| Cow_22 |  | 5 days | Lesion swab | No C_T_ | N/A | N/A | N/A | N/A | No C_T_ |
|  |  |  | Serum | No C_T_ | N/A | N/A | N/A | N/A | No C_T_ |
| Cow_23 |  | NCS | Serum | No C_T_ | N/A | N/A | N/A | N/A | No C_T_ |
| Cow_24 |  | 10 days | Serum | No C_T_ | N/A | N/A | N/A | N/A | No C_T_ |
| Cow_25 |  | 6 days | Lesion swab | 29.00 | No C_T_ | No C_T_ | No C_T_ | No C_T_ | 34.90 |
|  |  |  | Serum | No C_T_ | N/A | N/A | N/A | N/A | No C_T_ |
| Cow_26 |  | 10 days | Serum | No C_T_ | N/A | N/A | N/A | N/A | No C_T_ |
| Cow_27 |  | 7 days | Serum | No C_T_ | N/A | N/A | N/A | N/A | No C_T_ |
| Cow_28 |  | NCS | Serum | No C_T_ | N/A | N/A | N/A | N/A | No C_T_ |
| Cow_29 |  | 2 days | Epithelium | 17.35 | No C_T_ | 16.90 | No C_T_ | No C_T_ | 21.21 |
|  |  |  | Lesion swab | 30.80 | No C_T_ | No C_T_ | No C_T_ | No C_T_ | 39.14 |
|  |  |  | Serum | 36.90 | No C_T_ | No C_T_ | No C_T_ | No C_T_ | No C_T_ |
| Cow_30 | Morogoro Region,  Tanzania  (Farm 5) | ca. 1 month | Lesion swab | No C_T_ | N/A | N/A | N/A | N/A | No C_T_ |
|  |  |  | Serum | No C_T_ | N/A | N/A | N/A | N/A | N/A |
| Cow_31 |  | ca. 1 month | Serum | No C_T_ | N/A | N/A | N/A | N/A | N/A |
|  |  |  | OP fluid | No C_T_ | N/A | N/A | N/A | N/A | No C_T_ |
| Cow_32 |  | ca. 2 weeks | Lesion swab | No C_T_ | N/A | N/A | N/A | N/A | No C_T_ |
|  |  |  | Serum | No C_T_ | N/A | N/A | N/A | N/A | N/A |
| Cow_33 |  | ca. 1 month | Lesion swab | No C_T_ | N/A | N/A | N/A | N/A | No C_T_ |
|  |  |  | Serum | No C_T_ | N/A | N/A | N/A | N/A | N/A |
| Cow_34 |  | ca. 2 weeks | Serum | No C_T_ | N/A | N/A | N/A | N/A | N/A |
|  |  |  | OP fluid | No C_T_ | N/A | N/A | N/A | N/A | No C_T_ |
| Cow_35 |  | ca. 2 weeks | Lesion swab | No C_T_ | N/A | N/A | N/A | N/A | No C_T_ |
|  |  |  | Serum | No C_T_ | N/A | N/A | N/A | N/A | N/A |
| Cow_36 |  | ca. 1 month | Serum | No C_T_ | N/A | N/A | N/A | N/A | N/A |
|  |  |  | OP fluid | No C_T_ | N/A | N/A | N/A | N/A | No C_T_ |
| Cow_37 | Morogoro Region,  Tanzania  (Farm 6) | ca. 1-2 months | Serum | No C_T_ | N/A | N/A | N/A | N/A | N/A |
|  |  |  | OP fluid | 35.95 | No C_T_ | No C_T_ | No C_T_ | No C_T_ | 32.66 |
| Cow_38 |  | ca. 1-2 months | Serum | No C_T_ | No C_T_ | No C_T_ | No C_T_ | No C_T_ | N/A |
|  |  |  | OP fluid | No C_T_ | N/A | N/A | N/A | N/A | No C_T_ |
| Cow_39 |  | ca. 1-2 months | Serum | No C_T_ | No C_T_ | No C_T_ | No C_T_ | No C_T_ | N/A |
|  |  |  | OP fluid | No C_T_ | N/A | N/A | N/A | N/A | No C_T_ |
| Cow_40 |  | ca. 1-2 months | Lesion swab | No C_T_ | N/A | N/A | N/A | N/A | No C_T_ |
|  |  |  | Serum | No C_T_ | No C_T_ | No C_T_ | No C_T_ | No C_T_ | N/A |
| Cow_41 |  | ca. 1-2 months | Serum | No C_T_ | No C_T_ | No C_T_ | No C_T_ | No C_T_ | N/A |
|  |  |  | OP fluid | No C_T_ | N/A | N/A | N/A | N/A | No C_T_ |
| Cow_42 |  | ca. 1-2 months | Serum | No C_T_ | N/A | N/A | N/A | N/A | N/A |
|  |  |  | OP fluid | 32.65 | No C_T_ | No C_T_ | No C_T_ | No C_T_ | 30.44 |
| Cow_43 |  | ca. 1-2 months | Serum | No C_T_ | N/A | N/A | N/A | N/A | N/A |
|  |  |  | OP fluid | No C_T_ | N/A | N/A | N/A | N/A | No C_T_ |
| Cow_44 | Morogoro Region,  Tanzania  (Farm 7) | ca. 2 weeks | Lesion swab | No C_T_ | N/A | N/A | N/A | N/A | 38.31 |
|  |  |  | Serum | No C_T_ | N/A | N/A | N/A | N/A | No C_T_ |
| Cow_45 |  | 5 days | Epithelium | 28.60 | No C_T_ | 28.25 | No C_T_ | No C_T_ | 20.10 |
|  |  |  | Lesion swab | 32.25 | No C_T_ | No C_T_ | No C_T_ | No C_T_ | 25.37 |
|  |  |  | Serum | No C_T_ | N/A | N/A | N/A | N/A | 37.24 |
| Cow_46 |  | 4 days | Epithelium | 26.25 | No C_T_ | 23.45 | No C_T_ | No C_T_ | 17.05 |
|  |  |  | Lesion swab | 24.75 | No C_T_ | 22.30 | No C_T_ | No C_T_ | 18.83 |
|  |  |  | Serum | 37.50 | No C_T_ | No C_T_ | No C_T_ | No C_T_ | 27.57 |
| Cow_47 |  | 4 days | Epithelium | 23.75 | No C_T_ | 20.20 | No C_T_ | No C_T_ | 18.62 |
|  |  |  | Lesion swab | 23.30 | No C_T_ | 21.05 | No C_T_ | No C_T_ | 18.67 |
|  |  |  | Serum | No C_T_ | N/A | N/A | N/A | N/A | 38.03 |
| Cow_48 |  | 2 days | Epithelium | 19.85 | No C_T_ | 17.95 | No C_T_ | No C_T_ | 17.77 |
|  |  |  | Serum | 35.70 | No C_T_ | No C_T_ | No C_T_ | No C_T_ | 35.10 |
| Cow_49 |  | NCS | Serum | No C_T_ | N/A | N/A | N/A | N/A | N/A |
|  |  |  | OP fluid | No C_T_ | N/A | N/A | N/A | N/A | N/A |
| Cow_50 |  | NCS | Mouth swab | No C_T_ | N/A | N/A | N/A | N/A | No C_T_ |
|  |  |  | Serum | No C_T_ | N/A | N/A | N/A | N/A | N/A |
|  |  |  | OP fluid | No C_T_ | N/A | N/A | N/A | N/A | N/A |
| Cow­_51 |  | NCS | Serum | No C_T_ | N/A | N/A | N/A | N/A | N/A |
|  |  |  | OP fluid | No C_T_ | N/A | N/A | N/A | N/A | N/A |
| Cow_52 | Morogoro Region,  Tanzania  (Farm 8) | ca. 1-2 months | Lesion swab | No C_T_ | No C_T_ | No C_T_ | No C_T_ | No C_T_ | No C_T_ |
|  |  |  | Serum | No C_T_ | N/A | N/A | N/A | N/A | N/A |
|  |  |  | OP fluid | No C_T_ | N/A | N/A | N/A | N/A | N/A |
| Cow_53 |  | NCS | Serum | No C_T_ | No C_T_ | No C_T_ | No C_T_ | No C_T_ | N/A |
|  |  |  | OP fluid | No C_T_ | N/A | N/A | N/A | N/A | N/A |
| Cow_54 |  | NCS | Serum | No C_T_ | No C_T_ | No C_T_ | No C_T_ | No C_T_ | N/A |
|  |  |  | OP fluid | No C_T_ | N/A | N/A | N/A | N/A | N/A |
| Cow_55 |  | NCS | Serum | No C_T_ | N/A | N/A | N/A | N/A | N/A |
|  |  |  | OP fluid | No C_T_ | N/A | N/A | N/A | N/A | N/A |
| Cow_56 |  | NCS | Mouth swab | No C_T_ | N/A | N/A | N/A | N/A | No C_T_ |
|  |  |  | Serum | No C_T_ | N/A | N/A | N/A | N/A | N/A |
|  |  |  | OP fluid | No C_T_ | N/A | N/A | N/A | N/A | N/A |
| Cow_57 |  | 7 days | Lesion swab | No C_T_ | N/A | N/A | N/A | N/A | 38.11 |
|  |  |  | Serum | No C_T_ | N/A | N/A | N/A | N/A | N/A |
| Cow_58 |  | NCS | Serum | No C_T_ | N/A | N/A | N/A | N/A | N/A |
|  |  |  | OP fluid | No C_T_ | N/A | N/A | N/A | N/A | N/A |
| Cow_59 | Morogoro Region,  Tanzania  (Farm 9) | 5 days | Lesion swab | 30.55 | No C_T_ | No C_T_ | No C_T_ | No C_T_ | 23.08 |
|  |  |  | Serum | No C_T_ | N/A | N/A | N/A | N/A | No C_T_ |
| Cow_60 |  | 9 days | Lesion swab | 36.00* | N/A | N/A | N/A | N/A | No C_T_ |
|  |  |  | Serum | No C_T_ | N/A | N/A | N/A | N/A | N/A |
| Cow_61 |  | 5 days | Epithelium | 24.10 | No C_T_ | 27.00 | No C_T_ | No C_T_ | 15.24 |
|  |  |  | Serum | No C_T_ | N/A | N/A | N/A | N/A | No C_T_ |
| Cow_62 |  | 5 days | Lesion swab | 33.50 | No C_T_ | No C_T_ | No C_T_ | No C_T_ | 30.83 |
|  |  |  | Serum | No C_T_ | N/A | N/A | N/A | N/A | No C_T_ |
| Cow_63 |  | 4 days | Epithelium | 36.30 | No C_T_ | No C_T_ | No C_T_ | No C_T_ | 34.97 |
|  |  |  | Lesion swab | 34.25 | No C_T_ | No C_T_ | No C_T_ | No C_T_ | 26.30 |
|  |  |  | Serum | No C_T_ | N/A | N/A | N/A | N/A | No C_T_ |
| Cow_64 |  | 7 days | Lesion swab | No C_T_ | N/A | N/A | N/A | N/A | No C_T_ |
|  |  |  | Serum | No C_T_ | N/A | N/A | N/A | N/A | No C_T_ |
| Cow_65 |  | 4 days | Epithelium | 27.65 | No C_T_ | 36.00 | No C_T_ | No C_T_ | 23.10 |
|  |  |  | Serum | No C_T_ | N/A | N/A | N/A | N/A | No C_T_ |
|  |  | 7 days | Epithelium | 29.90 | No C_T_ | 28.05 | No C_T_ | No C_T_ | 22.61 |
| Cow_66 |  | 7 days | Epithelium | 37.50* | N/A | N/A | N/A | N/A | No C_T_ |
|  |  |  | Serum | No C_T_ |  |  |  |  | N/A |
| Cow_67 | Morogoro Region,  Tanzania  (Farm 10) | NCS | Serum | No C_T_ | N/A | N/A | N/A | N/A | N/A |
| Cow_68 | Morogoro Region,  Tanzania  (Farm 11) | 4 days | Epithelium | 33.85 | No C_T_ | No C_T_ | No C_T_ | No C_T_ | 31.08 |
|  |  |  | Lesion swab | 29.20 | No C_T_ | No C_T_ | No C_T_ | No C_T_ | 21.26 |
|  |  |  | Serum | No C_T_ | N/A | N/A | N/A | N/A | No C_T_ |
| Cow_69 |  | 4 days | Epithelium | 34.95 | No C_T_ | No C_T_ | No C_T_ | No C_T_ | 31.48 |
|  |  |  | Serum | No C_T_ | N/A | N/A | N/A | N/A | No C_T_ |
| Cow_70 |  | 7 days | Serum | No C_T_ | N/A | N/A | N/A | N/A | No C_T_ |
|  |  |  | OP fluid | 35.20 | No C_T_ | No C_T_ | No C_T_ | No C_T_ | 26.11 |
| Cow_71 |  | 10 days | Serum | No C_T_ | N/A | N/A | N/A | N/A | No C_T_ |
|  |  |  | OP fluid | No C_T_ | N/A | N/A | N/A | N/A | No C_T_ |
| Cow_72 |  | 4 days | Epithelium | 24.75 | No C_T_ | 26.05 | No C_T_ | No C_T_ | 16.65 |
|  |  |  | Serum | No C_T_ | N/A | N/A | N/A | N/A | No C_T_ |
| Cow_73 | Adama,  Ethiopia  (Farm 12) | 2 days | Epithelium | 22.50 | No C_T_ | 21.70 | No C_T_ | No C_T_ | N/A |
|  |  | 5 days | Lesion swab | 29.15 | No C_T_ | 35.00 | No C_T_ | No C_T_ | N/A |
|  |  |  | Serum | No C_T_ | N/A | N/A | N/A | N/A | N/A |
|  |  |  | OP fluid | 29.70 | No C_T_ | 35.00 | No C_T_ | No C_T_ | N/A |
| Cow_74 |  | NCS | Serum | No C_T_ | N/A | N/A | N/A | N/A | N/A |
| Cow_75 |  | 10 days | OP fluid | 36.40 | No C_T_ | No C_T_ | No C_T_ | No C_T_ | N/A |
| Cow_76 | Adama,  Ethiopia  (Farm 13) | 6 days | Lesion swab | 32.05 | No C_T_ | No C_T_ | No C_T_ | No C_T_ | N/A |
| Cow_77 |  | 7 days | Lesion swab | 38.05 | No C_T_ | No C_T_ | No C_T_ | No C_T_ | N/A |
| Cow_78 |  | 7 days | OP fluid | No C_T_ | N/A | N/A | N/A | N/A | N/A |
| All results represent the average C_T_ across two real-time reverse transcription PCR (rRT-PCR) replicates  “*”represents replicates where one was positive and the other negative  NCS: no clinical signs (“lesion swabs” in these animals were taken by swabbing either the mouth or feet directly)  N/A: not applicable (test not performed)  OP fluid: oesophageal-pharyngeal fluid  Shaded rows represent positive T-COR^TM^ 8 pan-serotype specific results (blue: positive serotype-specific results for serotype A, red: positive serotype-specific results for serotype O, grey: did not serotype)  **The reference rRT-PCR was performed in laboratory settings within East Africa using extracted RNA (this was not possible in Ethiopia due to a lack of facilities). | | | | | | | | | |
